# Can we predict post-surgical improvement in functional stooping in lumbar spinal stenosis? Insights from oblique lumbar interbody fusion outcomes and radiologic predictors

**DOI:** 10.1186/s12891-025-08821-7

**Published:** 2025-07-09

**Authors:** Dong-Ho Kang, Jonghyuk Baek, Bong-Soon Chang, Sam Yeol Chang, Dongook Kim, Sanghyun Park, Hyoungmin Kim

**Affiliations:** 1 Department of Orthopedic Surgery, Spine Center, Samsung Medical Center, 81 Irwon-ro, Gangnam-gu, Seoul, Republic of Korea; 2 Department of Orthopedic Surgery, Seoul National University College of Medicine, 101 Daehangno, Jongno-gu, Seoul, Republic of Korea; 3 Department of Orthopedic Surgery, Seoul National University Hospital, 101 Daehangno, Jongno-gu, Seoul, Republic of Korea

**Keywords:** Lumbar spinal stenosis, Short-level fusion, Oblique lumbar interbody fusion, Sagittal imbalance, Functional stooping

## Abstract

**Background:**

Functional stooping, characterized by a forward-flexed lumbar posture in patients with lumbar spinal stenosis (LSS), serves as a compensatory mechanism aimed at alleviating pain by expanding the constricted spinal canal. Surgeons widely use the oblique lateral interbody fusion (OLIF) to treat patients with LSS, restoring segmental lordosis in index surgical level. In some patients with LSS, improvement of global sagittal imbalance occurs after short-level OLIF. it remains unclear whether this is predominantly due to segmental correction or the resolution of functional stooping. Therefore, this study aimed to evaluate the effect of functional stooping resolution and segmental correction on sagittal imbalance after short-level OLIF, and identifying predictors of presence or absence of preoperative functional stooping in LSS.

**Methods:**

A retrospective review was conducted on LSS patients who underwent single or two-level OLIF with preoperative C7 sagittal vertical axis (SVA) > 50 mm. The clinical and radiological factors were analyzed. Logistic regression and receiver operating characteristic curve analysis were conducted to identify factors associated with presence or abscence of preoperative functional stooping, and to establish predictive threshold values, respectively.

**Results:**

A total of 103 patients with a mean age of 71.6 ± 8.6 years were included. In patients with preoperative functional stooping, segmental correction at the index surgical level contributed to only 47.7% of the total change of lumbar lordosis (LL), whereas the change of lordosis in remnant mobile lumbar segments constituted 52.3% of the total change of LL. Preoperative thoracic kyphosis (TK) (OR [95% CI]: 1.037 [1.002–1.073]), and preoperative SVA (OR [95% CI]: 0.986 [0.972–0.999]) were significant associated factors for predicting LSS patients without functional stooping.

**Conclusions:**

Functional stooping resolution markedly impacts global sagittal balance correction in LSS patients following short-level OLIF. Preoperative functional stooping correlates with greater TK and reduced SVA. For patients likely to achieve functional stooping resolution, single-level surgery may suffice initially, with deformity correction reserved if needed.

**Supplementary Information:**

The online version contains supplementary material available at 10.1186/s12891-025-08821-7.

## Background

Oblique lateral interbody fusion (OLIF) potentially offers superior advantages for lumbar lordosis (LL) restoration because of the use of a larger-sized cage than posterior lateral interbody fusion (PLIF) and transforaminal lateral interbody fusion (TLIF) [1–6]. While OLIF outperforms TLIF and PLIF in corrective capabilities, the degree of correction per level typically does not exceed 10° in the absence of anterior column realignment (ACR) or posterior osteotomy [7]. Importantly, addressing sagittal imbalance in short-segment fusion is crucial. Optimizing pelvic tilt reduction, enhancing C7 sagittal vertical axis (SVA), and aligning pelvic incidence (PI) with LL can potentially reduce postoperative complications, such as lower back and lower extremity pain or adjacent segment disease [8–10]. Current research on factors affecting sagittal imbalance after short-segment fusion or decompression surgery shows varied findings [11–16]. Fujii et al. found that higher stenotic levels correlate with decreased thoracic kyphosis (TK) [15], while Shin et al. observed that lower TK poses a risk for inadequate SVA normalization after decompression surgery [13].

The correction of global sagittal imbalance after surgery for lumbar spinal stenosis (LSS) is closely linked to ‘functional stooping.’ Previous studies have demonstrated recovery of LL and improvement in SVA following the resolution of preoperative functional stooping [12, 13, 17, 18]. This posture is understood as a compensatory mechanism in patients with neurogenic intermittent claudication, resulting from ligamentum flavum buckling, aimed at expanding the constricted spinal canal. When patients with LSS exhibit a forward-flexed lumbar posture or positive SVA (sagittal imbalance is defined as SVA ≥ 50 mm), it indicates an attempt to alleviate pain. The decompression of the dural sac with or without short-level fusion could correct this forward-bending posture.

Predicting postoperative improvement in functional stooping could guide the initial choice of decompression or short-level fusion over deformity surgery in patients with global sagittal imbalance. However, no research has quantitatively assessed the recuperation of LL and the enhancement of SVA via the resolution of preoperative functional stooping posture after short-level OLIF. The preoperative identification of a functional stooping component is crucial in determining the necessity of additional ACR or posterior osteotomy for achieving an ideal SVA if adequate LL restoration is not achieved through segmental correction during short-level OLIF. This study explored the respective influences of functional stooping resolution and segmental correction on sagittal imbalance correction after short-level OLIF and investigated the associated preoperative clinical and radiological factors to identify the absence or existence of functional stooping posture in LSS patients.

## Materials and methods

### Patients

We conducted a retrospective analysis of patients with LSS who underwent either single- or two-level minimally invasive OLIF (MIS-OLIF) from August 2012 to March 2023. The institutional review board of Seoul national university hospital approved the current study and the requirement for informed consent was waived because of its’ retrospective nature (IRB number: 2306-148-1441). The inclusion criteria were (1) patients who underwent either single- or two-level MIS-OLIF complemented with percutaneous pedicle screw instrumentation, (2) those with severe central stenosis (Schizas grade C or D) on preoperative MRI, and (3) patients with preoperative C7 SVA > 50 mm. The exclusion criteria were (1) patients who underwent additional open posterior decompression; (2) patients without 3 months postoperative whole spine lateral X-ray; (3) patients with a final fused level > 3; (4) patients with congenital hip dysplasia; (5) patients with concurrent compression fracture at the timing of OLIF; and (6) patients with congenital stenosis, malignancy, inflammatory disease, or infection.

### Surgery

During the study duration, OLIF was the primary surgical choice for LSS requiring fusion, irrespective of LSS severity. Direct decompressive procedures like PLIF and TLIF were reserved for patients with specific conditions such as sequestered intervertebral disc causing clear neurological deficits, prior retroperitoneal surgeries, or vascular obstructions at the L5-S1 disc level for surgeries at that level.

Most patients were treated with MIS-OLIF by using an anterolateral retroperitoneal approach while in the lateral decubitus position. Our goal was to maximize disc height without resorting to ACR or posterior osteotomy. A polyether–ether–ketone intervertebral cage packed with demineralized bone matrix was placed into the intervertebral space post-discectomy to facilitate the increase in posterior disc space height and foraminal height. In all cases, intraoperative C-arm imaging confirmed a significant increase in posterior disc space and foraminal heights. Cages with lordotic angles of 6° or 12° were implanted in all patients, positioned between the middle and anterior third of the disc space. Thereafter, patients were repositioned into a prone posture for the insertion of posterior percutaneous pedicle screws via intraoperative fluoroscopy. In the prone position, optimal lumbar sagging and maximal LL were achieved in index surgical levels using a chest bar and two posts under the anterior superior iliac spines; screws were inserted in situ without employing compression or distraction techniques.

### Criteria for functional stooping: defining I group and N group

**Fig. 1:**
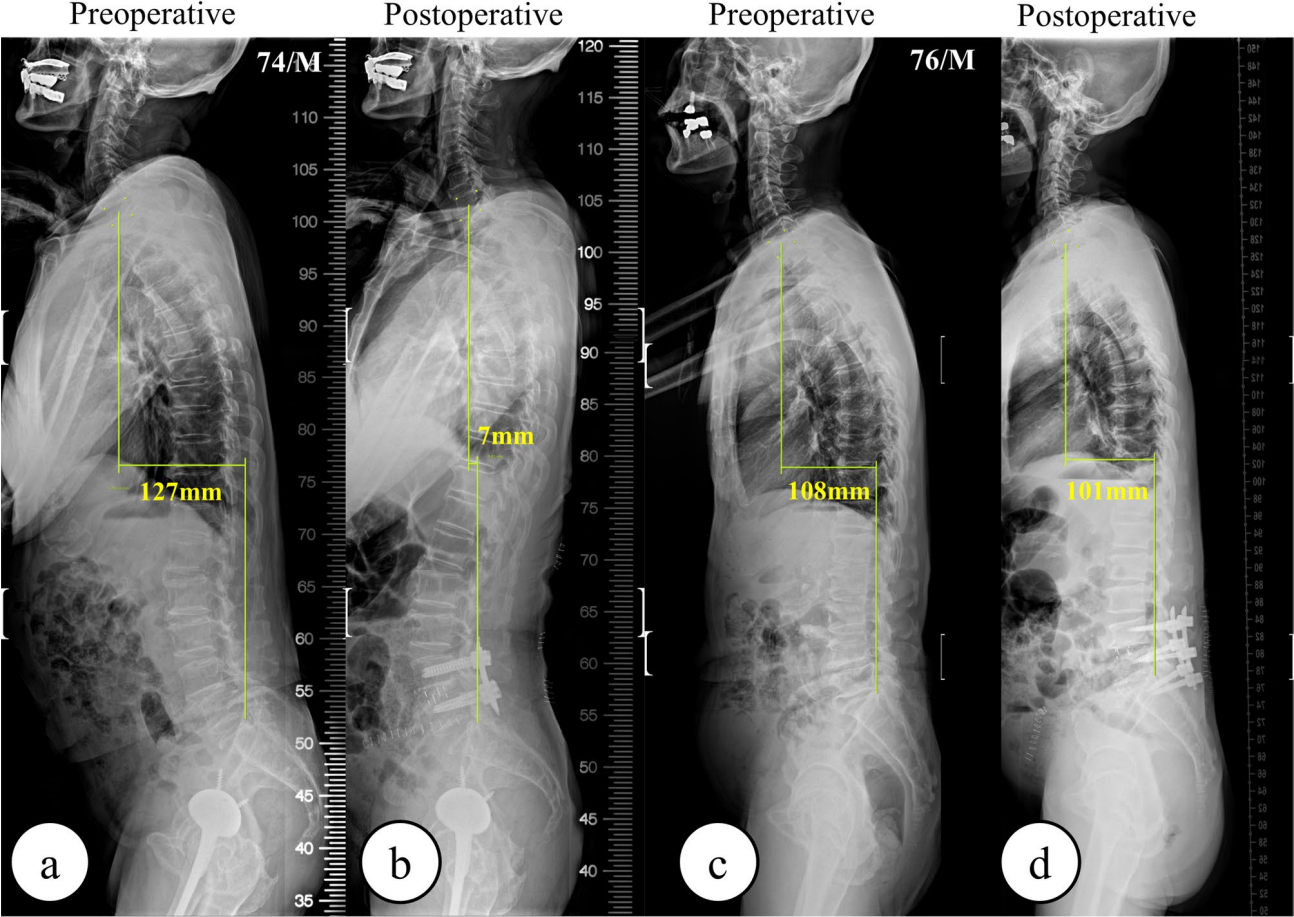
Comparative X-ray images of I and N group patients. (**a**) Preoperative and (**b**) postoperative whole spine lateral X-rays in the I group demonstrate significant correction of functional stooping in non-fused lumbar segments, indicated by changes in C7 SVA. (**c**) Preoperative and (**d**) postoperative X-rays in the N group reveal minimal improvement in lumbar lordosis in non-fused lumbar segments. SVA; Sagittal Vertical Axis

The presence of functional stooping posture and improvement of functional stooping after OLIF was determined using two criteria with preoperative and postoperative X-ray images: (1) a positive change of lordosis in remnant lumbar segments that were not fused, i.e., the change in LL minus the sum of changes in segmental angles (SAs) should exceed zero; (2) an increment in the segmental lordotic angle immediately superior to the surgical site postoperatively, i.e., the amount of change in the upper segmental angle (USA) after surgery should surpass zero. The first criterion was the calculation of the change in LL in non-fused segments. This was done by subtracting the sum of changes in SAs at the fused levels from the total change in LL. A positive result indicates an increase in lordosis in non-fused lumbar segments, reflecting functional stooping improvement. The second criterion was an increment in the segmental angle in USA. A positive change in USA indicated the resolution of the intentional lumbar flexion associated with functional stooping posture and reflected restored alignment in adjacent lumbar segments. Conversely, in cases where functional stooping was absent, OLIF surgery consistently increases segmental lordosis at the index level, and this is accompanied by reciprocal lordosis reduction in the adjacent, non-fused segments. Patients meeting both criteria were categorized into the improved functional stooping group (I group), whereas those without such improvement were assigned to the no functional stooping group (N group). Figure 1 shows the examples of patients in the I and N groups.

### Clinical data assessment

We extracted demographic and clinical data including age, sex, body mass index (BMI), concomitant spondylolisthesis, concomitant spondylolysis, number of surgical levels, exact level of fusion, and history of previous surgery at the index level from electronic medical records and the picture archiving and communication system. To evaluate the effectiveness of indirect decompression through OLIF, assessments including the Visual Analog Scale (VAS) for back and leg pain, along with the Oswestry Disability Index (ODI), were conducted before surgery and three months afterward.

### Radiological assessment

**Fig. 2:**
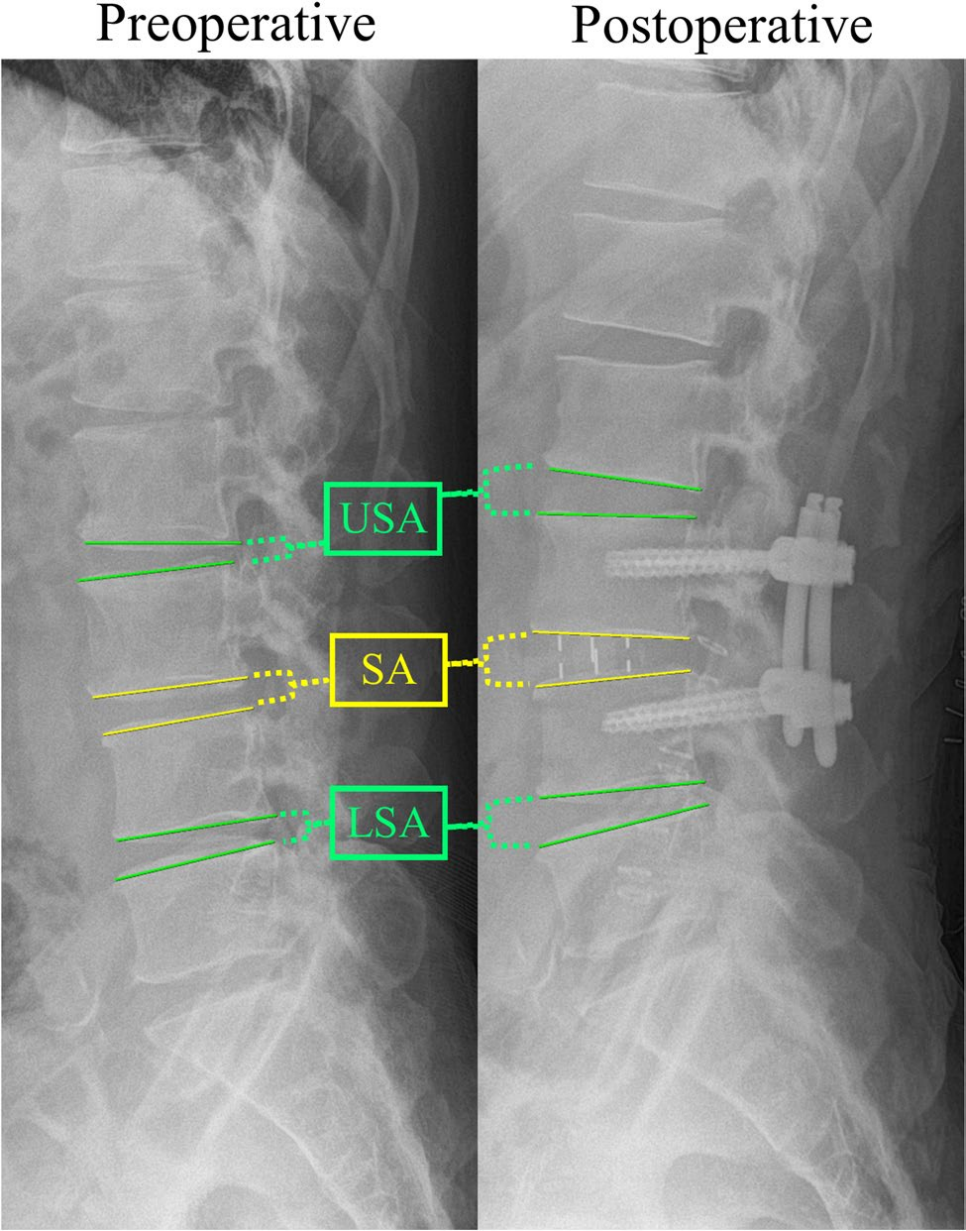
Radiological measurements. SA, segmental angle; USA, upper segmental angle; LSA, lower segmental angle

Regarding the radiological assessment, preoperative and 3-month postoperative lumbosacral and whole spine standing X-ray images and preoperative axial and sagittal T1- and T2-weighted magnetic resonance imaging (MRI) images were reviewed. To ensure accurate assessment of habitual postural alignment, all patients were instructed to stand for at least 10 min prior to the X-ray examination. Preoperative plain X-ray images were evaluated for various parameters including disc height, spondylolisthesis, angular instability, translational instability, and spondylolysis in each index surgical level. Instability was characterized by a range of motion exceeding 10°  at disc space or a translation of the vertebral body greater than 4 mm anteriorly and 2 mm posteriorly. We also investigated the preoperative and postoperative SA at the index surgical level; the USA, which is the SA above the upper instrumented vertebra; and the lower segmental angle (LSA) which is the SA below the lower instrumented vertebra (Fig. 2). Additionally, the following preoperative and postoperative sagittal measurements were assessed: PI, LL between the upper endplate of L1 and upper endplate of S1, sacral slope, pelvic tilt, TK between the upper endplate of T5 and lower endplate of T12, and C7 SVA.

In preoperative MRI images, we investigated several radiological characteristics in index surgical level including the osteoarthritis grading of facet joints [19], presence of facet cyst, presence of facet effusion [20], and Goutallier grade of the paraspinal muscle in the L5–S1 disc level [21]. The maximal central and foraminal stenoses in index surgical level were assessed with Schizas grade and Lee grade, respectively [22, 23]. Maximal central stenosis and foraminal stenosis were defined as the most severe stenotic lesions in the central and foraminal stenoses, respectively.

### Statistical analysis

Differences in continuous variables between the I and N groups were analyzed using Student’s t-test or the Kruskal–Wallis test, and categorical variables were evaluated using the chi-squared test, Fisher’s exact test, or linear-by-linear association. Logistic regression analysis was used to identify the preoperative factors associated to stooping improvement after OLIF. Variables showing a significant association (*p* < 0.20) in the univariate logistic regression analysis were incorporated into the multivariate logistic regression model. The backward elimination method was applied to compute odds ratios (ORs) and 95% confidence intervals (CIs) for the predictors of stooping improvement. Receiver operating characteristic (ROC) curves were employed to determine the area under the curve (AUC), which measures the sensitivity and specificity of identified risk factors in predicting stooping improvement. An AUC of 0.5–0.7 indicated no or low discriminatory power, 0.7–0.9 suggested moderate discriminatory power, and > 0.9 represented high discriminatory power. All statistical analyses were performed using IBM SPSS Statistics version 25.0 (IBM Corp., Armonk, NY, USA).

## Results

**Fig. 3:**
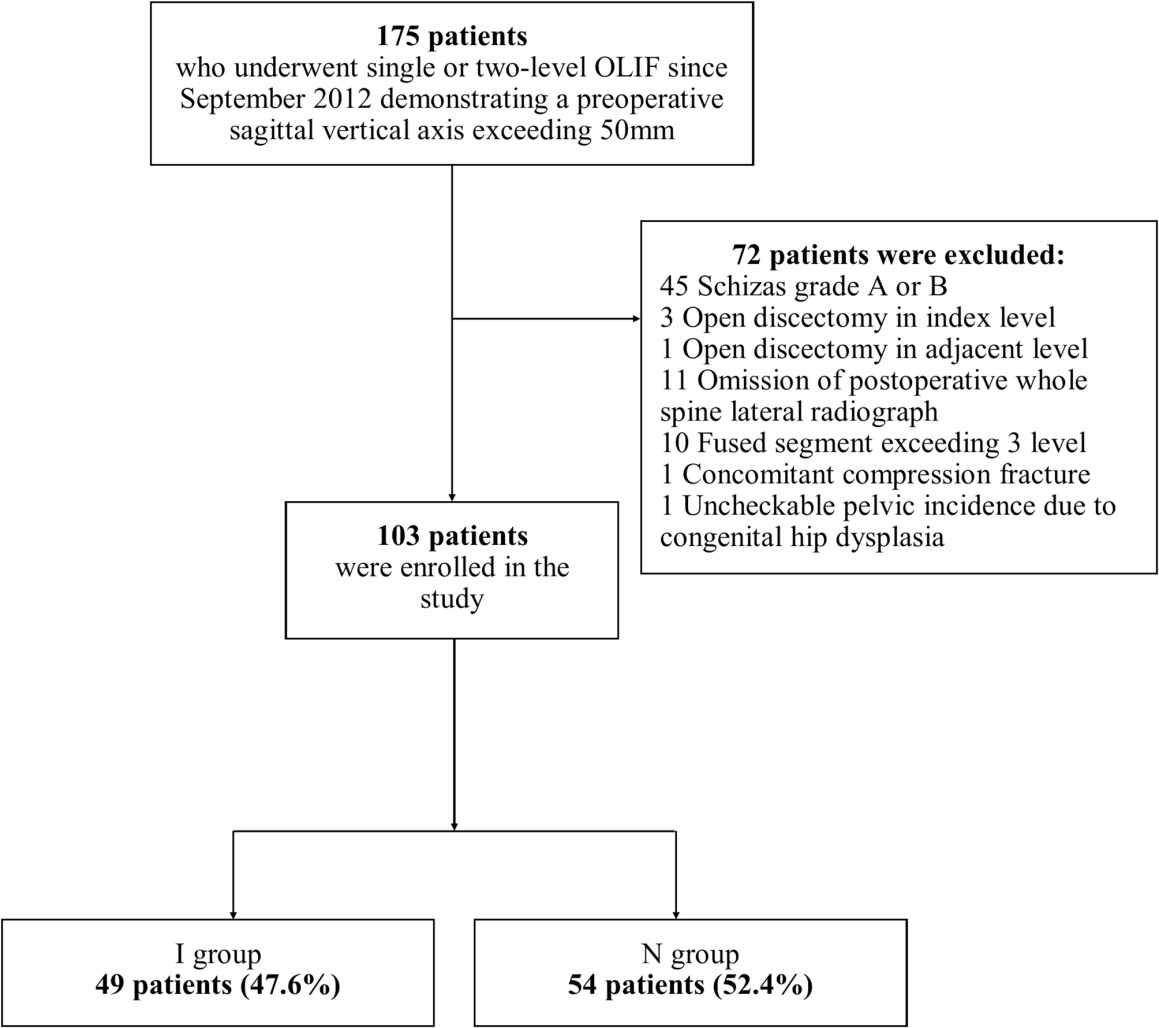
Flowchart of subject recruitment. OLIF, oblique lumbar interbody fusion

Our study enrolled a total of 103 patients with a mean ± standard deviation (SD) age of 71.6 ± 8.6 years (Fig. 3). Among these patients, 49 (47.6%) and 54 (52.4%) patients were assigned to the I group and N group, respectively. Table 1 outlines the baseline characteristics of the I and N groups and revealed that there were no statistically significant differences in age, sex, BMI, the proportion of patients with concomitant spondylolisthesis or spondylolysis, angular and translational instability, decreased disc height, the number of surgical levels, or precise surgical levels between the two groups. Among the patients, 65 patients (63.1%) showed an improvement of C7 SVA under 50 mm after surgery. No significant differences were observed between Group I and Group N in terms of the proportion of patients with a postoperative C7 SVA less than 50 mm, preoperative and 3-month postoperative VAS scores for back and leg pain, and preoperative and 3-month postoperative ODI scores.

**Table 1:** Baseline characteristics of included patients according to presence of preoperative functional stooping posture

Baseline characteristics	All patients		
	I group (*n* = 49)	N group (*n* = 54)	*P*-value
Age (mean ± SD, years)	70.3 ± 9.0	72.7 ± 8.0	0.078
Sex, Male (n [%])	13 [26.5%]	23 [42.6%]	0.088
BMI (mean ± SD, kg/m^2^)	26.4 ± 6.5	24.6 ± 6.8	0.085
Spondylolisthesis in index surgical level (n [%])	35 [71.4%]	43 [79.6%]	0.332
Two-level spondylolisthesis in index surgical level (n [%])	5 [10.2%]	6 [11.1%]	0.882
Spondylolysis in index surgical level (n [%])	0 [0.0%]	1 [1.9%]	> 0.999
Angular instability in index surgical level (n [%])	4 [8.2%]	5 [9.3%]	0.844
Translational instability in index surgical level (n [%])	2 [4.1%]	3 [5.6%]	0.728
Decreased disc height (n [%])	37 [75.5%]	41 [75.9%]	0.961
History of previous surgery in index surgical level (n [%])	5 [10.2%]	4 [7.4%]	0.733
Two-level OLIF (n [%])	19 [38.8%]	29 [53.7%]	0.129
Surgical level (n [%])			
L2-3	1 [2.0%]	1 [1.9%]	
L2-4	1 [2.0%]	1 [1.9%]	0.906
L3-4	3 [6.1%]	2 [3.7%]	
L3-5	13 [26.5%]	18 [33.3%]	
L4-5	25 [51.0%]	22 [40.7%]	
L4-S1	5 [10.2%]	9 [16.7%]	
L5-S1	1 [2.0%]	1 [1.9%]	
Postoperative C7 SVA < 50 mm (n [%])	34 [69.4%]	31 [57.4%]	0.208
Preoperative VAS for back pain (mean ± SD)	6.7 ± 1.9	6.4 ± 2.7	0.280
Preoperative VAS for leg pain (mean ± SD)	7.8 ± 1.7	7.7 ± 2.1	0.439
Preoperative ODI (mean ± SD)	37.9 ± 7.3	36.2 ± 8.3	0.164
3-month postoperative VAS for back pain (mean ± SD)	3.3 ± 2.3	3.0 ± 2.1	0.280
3-month postoperative VAS for back pain (mean ± SD)	2.7 ± 2.5	2.4 ± 2.5	0.295
3-month postoperative ODI (mean ± SD)	24.1 ± 6.5	25.2 ± 7.7	0.260

I group, functional stooping group; N group, no functional stooping group; SD, standard deviation; BMI, body mass index; OLIF, oblique lumbar interbody fusion; SVA, sagittal vertical axis; VAS, visual analog scale; SD, standard deviation; ODI, Oswestry disability index

Table 2 shows the comparison of radiological parameters in preoperative and postoperative X-ray images between the I and N groups. The I group showed significantly smaller preoperative LL (*p* = 0.042), preoperative TK (*p* = 0.010), postoperative TK (*p* = 0.041), preoperative USA (*p* = 0.022), and postoperative USA (*p* = 0.027) compared with the N group. The I group showed a significantly larger preoperative PI minus LL (*p* = 0.004), preoperative PT (*p* = 0.035), preoperative SVA (*p* = 0.009), total change in LL (*p* < 0.001), and change of lordosis in remnant lumbar segments that were not fused (*p* < 0.001) than the N group. Significantly greater improvements in LL after OLIF were observed in the I group than in the N group (mean ± SD; 13.0° ± 7.2° versus 7.0° ± 8.6°, *p* < 0.001). Conversely, the I group showed a lesser sum of changes in SA than the N group (mean ± SD; 6.2° ± 6.5° versus 8.6° ± 8.5°), however, the difference did not reach statistical significance (*p* = 0.053). Notably, in the I group, the SA change at the index surgical level constituted 47.7% of the total change of LL, whereas the change of lordosis in remnant lumbar segments that were not fused constituted 52.3% of the total change of LL. By contrast, the N group showed the sum of changes in SA that was larger than the total change in LL at the index surgical level. In the preoperative MRI, all radiologic parameters showed no significant difference between two groups (Table 3).

**Table 2:** Radiologic parameters of patients grouped by the presence of preoperative functional stooping posture. Values are presented as degrees (mean ± SD) for each parameter

Radiologic parameters	All patients		
	I group (*n* = 49)	N group (*n* = 54)	*P*-value
Preoperative PI	56.1 ± 10.4	53.5 ± 10.3	0.097
Postoperative PI	56.2 ± 10.4	53.4 ± 10.3	0.089
Preoperative LL	30.7 ± 13.5	35.4 ± 13.5	0.042
Postoperative LL	43.7 ± 10.8*	42.3 ± 10.0*	0.254
Preoperative PI - LL	25.4 ± 14.2	18.1 ± 13.4	0.004
Postoperative PI - LL	12.5 ± 10.2*	11.1 ± 10.0*	0.242
Preoperative PT	25.7 ± 9.1	22.4 ± 8.9	0.035
Postoperative PT	21.2 ± 8.6*	20.3 ± 8.2*	0.296
Preoperative SS	30.5 ± 8.4	31.6 ± 8.5	0.244
Postoperative SS	35.0 ± 7.6*	33.1 ± 7.3*	0.104
Preoperative TK	29.4 ± 12.0	35.1 ± 12.4	0.010
Postoperative TK	34.4 ± 11.6*	38.3 ± 10.9*	0.041
Preoperative SVA	93.5 ± 36.1	78.8 ± 25.8	0.009
Postoperative SVA	34.6 ± 36.7*	43.4 ± 34.8*	0.108
Preoperative USA	7.6 ± 4.1	9.3 ± 4.2	0.022
Postoperative USA	10.7 ± 4.2*	9.1 ± 4.2	0.027
Preoperative LSA	11.4 ± 5.9	12.6 ± 6.8	0.181
Postoperative LSA	12.7 ± 5.3*	13.3 ± 6.1	0.323
Δ LL	13.0 ± 7.2	7.0 ± 8.6	< 0.001
Sum of Δ SA	6.2 ± 6.5	8.6 ± 8.5	0.053
Δ LL - Sum of ΔSA	6.8 ± 4.7	-1.7 ± 8.0	< 0.001

* *P* < 0.05 compared with preoperative

SD, standard deviation; I group, functional stooping group; N group, no functional stooping group; PI, pelvic incidence; LL, lumbar lordosis; PT, pelvic tilt; SS, sacral slope; TK, thoracic kyphosis; SVA, sagittal vertical axis; USA, upper segmental angle; LSA, lower segmental angle; SA, segmental angle

**Table 3:** Radiologic characteristics of the included patients on preoperative MRI

Radiologic characteristics	All patients		
	I group (*n* = 49)	*N* group (*n* = 54)	*P*-value
Sequestrated disc in index surgical level (n[%])	13 [26.5%]	8 [14.8%]	0.141
Facet effusion in index surgical level (n [%])	33 [67.3%]	33 [61.1%]	0.510
Facet cyst in index surgical level (n [%])	2 [4.1%]	2 [3.7%]	> 0.999
Grade 3 facet osteoarthritis in index surgical level (n [%])	30 [61.2%]	30 [55.6%]	0.560
Goutallier grade of paraspinal muscle in L5-S1 level (n [%])			
Grade 0	2 [4.1%]	5 [9.3%]	0.892
Grade 1	15 [30.6%]	4 [7.4%]	
Grade 2	27 [55.1%]	43 [79.6%]	
Grade 3	2 [4.1%]	1 [1.9%]	
Grade 4	3 [6.1%]	1 [1.9%]	
Schizas grade of maximal central stenosis (n [%])			
Grade C	16 [32.7%]	17 [31.5%]	0.899
Grade D	33 [67.3%]	37 [68.5%]	
Lee grade of maximal foraminal stenosis (n [%])			
Grade 0	11 [22.4%]	4 [7.4%]	0.151
Grade 1	10 [20.4%]	11 [20.4%]	
Grade 2	4 [8.2%]	8 [14.8%]	
Grade 3	24 [49.0%]	31 [57.4%]	

MRI, magnetic resonance imaging; I group, functional stooping group; N group, no functional stooping group

In the multivariate logistic regression analyses, preoperative TK (OR [95% CI]: 1.037 [1.002–1.073]), and preoperative SVA (OR [95% CI]: 0.986 [0.972–0.999]) were significant associated factors for predicting patients without preoperative functional stooping posture (Table 4, full-version in the Appendix).

**Table 4:** Univariate and multivariate logistic regression analyses for predicting patients without preoperative functional stooping posture

	Univariate analysis		Multivariate analysis	
	Odds ratio (95% CI)	*P* value	Odds ratio (95% CI)	*P* value
Sex (male)		0.090		0.172
Preoperative LL		0.087		0.699
Preoperative PI minus LL	0.962 (0.934—0.991)	0.011		0.590
Preoperative PT		0.072		0.457
Preoperative TK	1.040 (1.005—1.075)	0.024	1.037 (1.002—1.073)	0.038
Preoperative SVA	0.985 (0.972—0.998)	0.023	0.986 (0.972—0.999)	0.036
Preoperative USA	1.103 (1.001—1.216)	0.049		0.458

CI, confidence interval; LL, lumbar lordosis; PI, pelvic incidence; PT, pelvic tilt; TK, thoracic kyphosis; SVA, sagittal vertical axis; USA, upper segmental angle

**Fig. 4:**
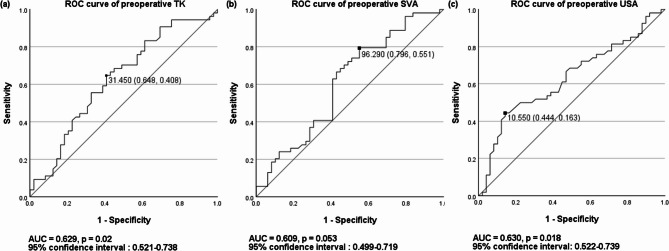
Receiver operating characteristic (ROC) curves illustrating the diagnostic accuracy of (**a**) preoperative SVA with a threshold value of 78.04°, (**b**) preoperative TK with a threshold value of 31.85°, and (**c**) preoperative USA with a threshold value of 10.55° in predicting patients without preoperative functional stooping posture. SVA, sagittal vertical axis; TK, thoracic kyphosis; USA, upper segmental angle; AUC, area under the ROC curve

The ROC curve analysis established threshold values for predicting patients without preoperative functional stooping posture. Preoperative TK (AUC: 0.629; 95% CI: 0.521–0.738; *p* = 0.020), preoperative SVA (AUC: 0.609; 95% CI: 0.499–0.719; *p* = 0.053), and preoperative USA (AUC: 0.630; 95% CI: 0.522–0.739; *p* = 0.018) demonstrated low discriminatory power (Fig. 4). Non-stooping tendency was likely in patients with preoperative TK > 31.5° and SVA between 50 and 96 mm (OR: 5.091; specificity: 0.59; positive predictive value: 0.63). Incorporating USA > 10.5° with TK > 31.5° and SVA between 50 and 96 mm enhanced the prediction of patients without preoperative functional stooping posture (OR: 11.719; specificity: 0.94; positive predictive value: 0.86). Patients with preoperative TK < 31.5° and USA < 10.5° were more likely to exhibit a functional stooping tendency (OR: 5.913; specificity: 0.74; positive predictive value: 0.63). The combination of USA < 10.5°, TK < 31.5°, and SVA > 96 mm enhanced the ability to predict preoperative functional stooping posture (OR: 2.415; specificity: 0.89; positive predictive value: 0.65).

## Discussion

Our study’s key findings demonstrate that patients lacking preoperative functional stooping can be predicted pre-surgery through a combination of preoperative X-ray parameters. Sagittal imbalance was ameliorated with an improvement of C7 SVA under 50 mm in approximately 65% of patients with LSS following short-level OLIF, and resolving preoperative functional stooping posture had a major effect on correction of global sagittal imbalance. Shin et al. reported 73% of normalization rate in SVA after decompression surgery [13]. Similarly, approximately 70% of patients in the I group exhibited a postoperative C7 SVA below 50 mm compared with 61% of patients in the N group. No significant differences were observed in preoperative and 3-month postoperative VAS for back pain, VAS for leg pain, and ODI scores between the two groups, with both groups demonstrating improvements in these clinical scores after surgery (Table 1). These findings indicate that the indirect decompression provided by OLIF effectively alleviated stenosis-related symptoms in both groups, which is aligned with Hiyama’s study [24].

The I group showed a more severe sagittal malalignment preoperatively with a larger preoperative PI minus LL and preoperative SVA than the N group. The change of total LL is significantly larger in the I group than in the N group. In the I group, the sum of changes in SA at the index surgical level contributed to only 47.7% of the total change of LL, whereas the change of lordosis in remnant lumbar segments that were not fused constituted 52.3% of the total change of LL. These findings indicate that surgeons may recommend unnecessarily aggressive surgical procedures, such as posterior osteotomy, for patients with severe sagittal imbalance and preoperative functional stooping. In these patients, there is a 70% likelihood that OLIF alone can correct preoperative sagittal imbalance. Previous studies have associated the adequate correction of segmental lordosis in index surgical level with good clinical outcomes [25, 26]. Our study showed that while segmental angle correction through OLIF is important in LSS patients for correction of sagittal imbalance, resolution of functional stooping in the mobile segment of the remaining lumbar spine also play a key role in rectifying sagittal imbalance.

In the N group, the restoration of LL was entirely attributable to the correction of the SA in the index surgical level. The sum of changes in SA was larger than the total change of LL, thus indicating a kyphotic change in the remnant lumbar segments that were not fused. Therefore, for patients in the N group, where improvement in lordosis due to the resolution of functional stooping within remnant lumbar segments that were not fused is unlikely, additional procedures such as ACR or posterior osteotomy may be necessary to achieve further lordosis restoration and to correct SVA. Costanzo showed that the average corrections per level after lateral lumbar interbody fusion were less than 10° without ACR or posterior osteotomy [7]. Furthermore, Li et al. showed that despite large cage placement during OLIF, the effect of OLIF in the index surgical level was insufficient to significantly modify overall spinal sagittal alignment [8]. Therefore, if patients who are expected to be allocated in the N group show no functional stooping posture preoperatively, surgeons should prepare additional procedures in the process of OLIF, such as ACR or posterior osteotomy, by considering the potential necessity for further correction of LL.

Concerning the identification of preoperative factors associated with improvement of functional stooping after OLIF, logistic regression reveals that a larger preoperative TK and relatively smaller SVA are significant predictors of patients without preoperative functional stooping posture. These findings could be explained by the involuntary functional stooping posture in lumbar segments, which is similar to the sciatic list observed in patients with pediatric herniated intervertebral disc. In these patients, a voluntary reduction in TK will compensate for the involuntary forward bending of the lumbar segment. This is echoed by Fujii, who demonstrated that patients with a higher stenotic level showed a significantly smaller TK [15]. Contrary to our result, Shin et al. reported that low TK was a risk factor for not achieving SVA normalization after decompression surgery [13]. They explained this result via the inclusion of patients with degenerative lumbar kyphosis who showed subnormal TK (16.3 ± 14.2°) to compensate for their sagittal imbalance of lumbar kyphosis. This discrepancy might originate from the difference in composition of included patients. In the current study, because we investigated only single- or two-level OLIF, no patient with degenerative lumbar kyphosis was included.

Our results showed that a relatively smaller SVA is a predictor of patients without preoperative functional stooping posture, thus suggesting that a larger SVA is related to the presence of preoperative functional stooping posture. Fujii et al. reported that the improvement in LL and SVA was greater in patients with poor preoperative alignment [15]. However, several studies showed that severe preoperative sagittal imbalance had residual imbalance after decompression surgery [13, 16]. This discrepancy could also be explained by the difference in the composition of our included patients from these studies, which included patients with degenerative lumbar kyphosis. When patients with LSS with concurrent degenerative lumbar kyphosis show severe preoperative sagittal imbalance, deformity correction surgery is likely needed to correct genuine sagittal deformity in addition to resolving functional stooping posture.

By using ROC curve analysis, we could provide measurable criteria for determining the necessity of additional ACR or posterior osteotomy for an optimal SVA in short-level OLIF. Patients with a preoperative upper USA > 10.5°, TK > 31.5°, and SVA between 50 and 96 mm, as evidenced in standing X-rays, have an 86% likelihood of not resolving functional stooping in remnant lumbar segments. Therefore, in patients with these conditions, additional procedures such as ACR or posterior osteotomy may be required during OLIF procedure if adequate LL restoration is not achieved through SA correction. On contrary, patients with a preoperative USA > 10.5°, TK < 31.5°, and SVA > 96 mm, as evidenced in standing X-rays, have an 86% likelihood of functional stooping in remnant lumbar segments. Therefore, in patients with these conditions, single-level decompression or fusion surgery may initially address stenosis-related symptoms. If stooping persists, deformity surgery can be considered as a second-stage procedure.

This study has several limitations. First, the criteria for judging the presence of preoperative functional stooping and improvement of functional stooping after OLIF was not clinically validated. However, no study has investigated the resolution of functional stooping quantitatively. Therefore, as an inaugural study in this domain, the current study may provide a reference point for future research. Second, owing to the retrospective nature of the study, the results may be subjected to a degree of uncertainty because of potential selection bias from excluded comorbidities. However, these exclusions are not expected to significantly impact our conclusions. Third, in this study, while the focus was on patients with an SVA exceeding 50 mm, we did not assess sagittal imbalance compensation in knee, hip, or ankle joints. However, when the clinical information of the patients was retrospectively reviewed, no patients were described with severe knee flexion contracture or severe hip flexion contracture. Given that our analysis is focused on the change of imaging parameters for each patient, the inclusion of such conditions is unlikely to impact the study’s result. Fourth, the evaluation of other clinical scores, including Japanese orthopaedic association back pain evaluation questionnaire, were not performed. However, this study originally aimed to find the associated preoperative clinical and radiological factors to identify the existence of correctable functional stooping posture after short-level OLIF. Therefore, the exclusion of other clinical scores was not anticipated to substantially alter the outcome of our analysis. Fifth, while this study does not address long-term clinical outcomes. Further studies with extended follow-up are needed to evaluate the durability and clinical impact of sagittal alignment correction. One other limitation is the absence of postoperative MRI in our analysis. Although postoperative MRI could have provided more definitive evidence of successful indirect decompression, it is not routinely obtained in clinical practice. However, the substantial improvements in clinical scores, including VAS and ODI, strongly support the effectiveness of the surgical intervention. We have limited our findings to preoperative MRI, which was sufficient for selecting patients with Schizas grade C and D stenosis. Future studies incorporating postoperative MRI could further validate the outcomes of indirect decompression and its impact on sagittal balance correction. Despite these limitations, this study presents a novel approach for identifying the effectiveness of short-level OLIF on sagittal imbalance and the influence of functional stooping posture and provides a comprehensive understanding of the preoperative factors associated with functional stooping improvement.

## Conclusion

The resolution of functional stooping posture significantly influences the correction of global sagittal imbalance in patients with LSS undergoing short-level OLIF. For patients showing severe sagittal imbalance with preoperative functional stooping, surgeons should exercise caution to avoid unnecessarily aggressive procedures aimed at correcting preoperative sagittal imbalance. A larger preoperative TK and a relatively smaller preoperative SVA are predictive factors associated with preoperative functional stooping. Patients with specific preoperative sagittal alignment characteristics may be less likely to achieve functional stooping resolution through OLIF alone. In such cases, additional procedures like anterior column realignment or posterior osteotomy may be necessary to achieve adequate lumbar lordosis restoration. Conversely, for patients whose preoperative alignment suggests a strong likelihood of functional stooping resolution, single-level decompression or fusion surgery may effectively address stenosis-related symptoms initially. Deformity correction surgery can then be reserved as a second-stage procedure if stooping persists. These findings highlight the importance of personalized surgical strategies tailored to preoperative radiological assessments to optimize outcomes.

## Electronic supplementary material

Below is the link to the electronic supplementary material.

Supplementary Material 1

## Data Availability

The datasets generated and/or analyzed during the current study are not publicly available due to patient privacy but are available from the corresponding author on reasonable request.

## Abbreviations

OLIF: Oblique lateral interbody fusion
LL: Lumbar lordosis
PLIF: Posterior lateral interbody fusion
TLIF: Transforaminal lateral interbody fusion
ACR: Anterior column realignment
SVA: Sagittal vertical axis
PI: Pelvic incidence
TK: Thoracic kyphosis
LSS: Lumbar spinal stenosis
MIS-OLIF: Minimally invasive OLIF
SAs: Segmental angles
USA: Upper segmental angle
BMI: Body mass index
VAS: Visual Analog Scale
ODI: Oswestry Disability Index
MRI: Magnetic resonance imaging
LSA: Lower segmental angle
ORs: Odds ratios
CIs: Confidence intervals
ROC: Receiver operating characteristic
AUC: Area under the curve
SD: Standard deviation
