# Supplementary material for: Can we predict post-surgical improvement in functional stooping in lumbar spinal stenosis? Insights from oblique lumbar interbody fusion outcomes and radiologic predictors
Source: BMC Musculoskelet Disord. 2025 Jul 9;26:670. doi: 10.1186/s12891-025-08821-7 (PMC12239410; doi:10.1186/s12891-025-08821-7)
Supplement: Supplementary file 1 — Supplementary Material 1 [file 12891_2025_8821_MOESM1_ESM.docx]

Appendix. Full version of univariate and multivariate logistic regression analyses for predicting patients without preoperative functional stooping posture.

|  | Univariate analysis | | Multivariate analysis | |
| --- | --- | --- | --- | --- |
|  | Odds ratio (95% CI) | *P* value | Odds ratio (95% CI) | *P* value |
| Age (years) |  | 0.161 |  |  |
| Sex (male) |  | 0.090 |  | 0.172 |
| BMI |  | 0.172 |  |  |
| 2-level OLIF ^a^ |  | 0.131 |  |  |
| Revision |  | 0.617 |  |  |
| Presence of spondylolytic spondylolisthesis in index surgical level |  | >0.999 |  |  |
| Preoperative PI |  | 0.194 |  |  |
| Preoperative LL |  | 0.087 |  | 0.699 |
| Preoperative PI minus LL | 0.962 (0.934—0.991) | 0.011 |  | 0.590 |
| Preoperative PT |  | 0.072 |  | 0.457 |
| Preoperative SS |  | 0.484 |  |  |
| Preoperative TK | 1.040 (1.005—1.075) | 0.024 | **1.037 (1.002—1.073)** | **0.038** |
| Preoperative SVA | 0.985 (0.972—0.998) | 0.023 | **0.986 (0.972—0.999)** | **0.036** |
| Preoperative RLL | 1.040 (1.007—1.074) | 0.017 |  |  |
| Preoperative LDI |  | 0.227 |  |  |
| Preoperative RPV |  | 0.063 |  | 0.356 |
| Preoperative GT | 0.956 (0.920—0.993) | 0.022 |  | 0.446 |
| Preoperative USA | 1.103 (1.001—1.216) | 0.049 |  | 0.458 |
| Preoperative LSA |  | 0.357 |  |  |
| Presence of decreased disc height |  | 0.961 |  |  |
| Presence of facet effusion in surgical level |  | 0.510 |  |  |
| Presence of bilateral facet effusion in surgical level |  | 0.856 |  |  |
| Presence of facet cyst |  | 0.921 |  |  |
| Presence of sequestrated disc |  | 0.145 |  |  |
| Presence of grade 3 facet arthropathy |  | 0.560 |  |  |
| Schizas grade D of maximal central stenosis |  | 0.899 |  |  |
| Lee grade 3 of maximal foraminal stenosis |  | 0.392 |  |  |
| Goutallier grade of paraspinal muscle in L5-S1 level ≥ 3 |  | 0.209 |  |  |
| ^a^ Odds compared to single level | | | | |

GAP score, global alignment and proportion score; BMI, body mass index; OLIF, oblique lumbar interbody fusion; LL, lumbar lordosis; PI, pelvic incidence; PT, pelvic tilt; SS, sacral slope; TK, thoracic kyphosis; SVA, sagittal vertical axis; SA, segmental angle; USA, upper segmental angle; LSA, lower segmental angle;
